# A study on mental health and its influencing factors among police officers during the COVID-19 epidemic in China

**DOI:** 10.3389/fpsyt.2023.1192577

**Published:** 2023-06-07

**Authors:** Ji Wu, Qiong Wu, Minghui Xia, Jing Xiao, Xin Yan, Dao Li

**Affiliations:** ^1^Faculty of Medicine, Wuhan City College, Wuhan, China; ^2^Hospital of Stomatology of Wuhan University, Wuhan, China; ^3^Xiantao Vocational College, Xiantao, China; ^4^Yangtze River Engineering Vocational College, Jingzhou, China

**Keywords:** mental health, police officers, COVID-19 pandemic, work stress, risk perception

## Abstract

**Background:**

The outbreak of the COVID-19 pandemic has had a tremendous impact on people’s health and well-being. The crisis also threw into sharp relief the fact that police officers faced an increased risk of developing mental health problems. The main purpose of this study was to explore the effects of work stress and risk perception on the mental health of police officers during the epidemic.

**Methods:**

We conducted a cross-sectional online survey among police officers in Wuhan city, China, and data were collected from 11 March to 12 May 2022. A total of 358 questionnaires were received, of which 302 were considered valid. The questionnaires included demographic information, work stress scale, Symptom Checklist 90 (SCL-90) and epidemic risk perception scale. Descriptive analyses, one-way analysis of variance and linear regression were used to analyze the data.

**Results:**

The prevalence of mental health problems was 38.74% among the surveyed police officers. The results indicated that the total score of SCL-90 and its subdimensions were positively correlated with work stress and risk perception. Moreover, we found that three factors were relevant to the police’s mental health: age, marital status, and education.

**Conclusion:**

Front-line police officers tend to show a higher prevalence of symptoms of mental disorders during the COVID-19 pandemic. We found that increased work stress and risk perception may adversely affect police officers’ mental health. Consequently, policy-makers and police organizations should establish an internal mental health problem coping team to improve police officers’ mental health resilience.

## Introduction

The sudden outbreak of the COVID-19 pandemic has had a tremendous impact worldwide. With the constant mutation of the virus, the escalation of public health emergencies has severely affected people’s normal lives globally. To maintain social stability, the police have engaged in maintaining social security and public order. In addition, they were responsible for helping the health administration carry out additional police work, such as patient isolation, home quarantine control, and transport of epidemic prevention materials (1, 2). When they perform their duties, they were challenged by the situation of the infected people and their own fears caused by COVID-19 pandemic, the police officers will face great psychological pressure of exposure to the virus (3). The rising number of infected or COVID-19-related fatalities among police officers is problematic. Community police are the largest number of police officers who stick to the front line of the prevention and control of epidemics in public security police stations (4).

As such, police officers may experience greater negative emotions, which can lead to more severe mental health issues. Attention has been devoted to the important role of police officers’ mental health. Tian Yong et al. noted that the positive detection rate of psychological problems among prison police was 54.63%, which was significantly higher than that of other people in China (5). This emerged COVID-19 pandemic has led to changes in the police’s routine activities. It even caused most police to suffer from fatigue caused by unconventional work tasks. According to Xinhuanet, a total of 22,930 police officers and more than 7,200 vehicles have been invested in Hubei Province to fight the pandemic (6). They were in long-term service, fully quarantined working mode, and delivered high-intensity emergency services. Approximately 73% of police officers reported a significant increase in work intensity and workload during the COVID-19 pandemic (7). Similarly, it is worth noting that about 99.31% of medical staff reported they faced high work risk and workload, and 28.60% of them worked more than 50 h per week in the COVID-19 pandemic (8). Under the extreme work pressure of epidemic prevention and control work, public security officers are faced with excessive exhaustion of psychological resources and physiological exhaustion. Based on transactional stress theory (9), stress results from the individual’s perception of a discrepancy between the subjective negative appraisal situation on him or her and his or her coping resource demands in a particular situation (10). The epidemic exacerbated police role conflict, and they became vulnerable to the psychological crisis. Specifically, the continued increase in stress at work may lead to emotional exhaustion, and it further affects individual’s chances of developing depressive symptoms (11).

Perceived risk may be defined as the perceived likelihood of personally encountering a hazard (12) and cognitive judgment of their susceptibility to risk. Although moderate risk perception contributes to raising people’s level of alertness, which is beneficial for individual survival and creation, it can also be exacerbated and amplified by intrusive catastrophic thoughts. The heuristic effect indicates that people react to the hazard, not only influenced by objective information but also on the basis of their direct feelings (13). Human beings might perceive risk as more threatening when they approach it gradually. For instance, life-threatening stimuli from a high-risk work environment have negatively impacted police. Police officers were fearful of being infected or even dying while helping to transport and quarantine confirmed patients during the pandemic. Thus, cognitive assessment and emotional judgments are often skewed by negative perception (14).

Although the prevalence of mental illness among officers has been documented, studies focusing on the mental health of police and its influencing mechanism in the COVID-19 epidemic still have a knowledge gap. A previous study was based on the psychological state of health care workers (15–17); unfortunately, the research on police officers only describes the level of change in their mental state but ignores the impact and boundary conditions of police officers’ special working characteristics. Therefore, to better understand the impact of work stress and perceived risk on the mental health of police officers in Wuhan city during the epidemic period, we conducted this survey to assess the prevalence of current symptoms of mental illness among the police and identify the psychological factors related to their mental health.

Based on the literature presented above, we formulated three hypotheses:

*H1*: Demographic characteristics are positively correlated with police officers’ mental health status.

*H2*: Work stress is positively correlated with mental health risk among police officers.

*H3*: The higher the risk perception of police officers is, the worse their mental health.

## Methods

### Study design and sample

A prospective cross-sectional questionnaire online survey was conducted from 11 March to 12 May 2022. It was the height of the fourth wave of the COVID-19 epidemic. In-service police officers in Wuhan city (Hankou, Wuchang, Hanyang districts) were selected, and questionnaires were conducted by using convenience and snowball sampling methods. After informed consent was granted, the participants completed the questionnaire on site, which took approximately 15 to 20 min to finish. Ethical guidelines were strictly followed, and the study protocol was scrutinized and approved by the institutional and national committee on Human Experimentation. Inclusion criteria were police officers who worked in Wuhan city, including security police, criminal police, constable police, community police, and internal work police. They were willing to participate in this investigation and complete the questionnaire with high quality. A total of 358 questionnaires were received in the study, of which 302 were considered valid (84.36%).

### Measures

#### Mental health assessment (SCL-90)

Symptom Checklist 90 (SCL-90) assesses psychological distress and symptoms of psychopathology. This scale covers 9 factors with a total of 90 questions (18). Specifically, they are somatization, obsessive–compulsive, interpersonal sensitivity, depression, anxiety, hostility, phobic anxiety, paranoid ideation, and psychosis. Positive items on the SCL-90 refer to the number of items with a single score above 2. Likewise, if the number of positive items exceeds 43 or the total score is more than 160, it can be considered positive (19). Higher total scores of the nine indicative factors corresponded with poorer mental health status. The Cronbach’s alpha value was 0.980, which indicates good internal consistency of the SCL-90.

#### Work stress

The work stress was measured using the validated measurement tool (20) in this study. A 14-item self-report questionnaire assessed police officers’ perceived stress in the workplace through three main dimensions: workload (the pressure of work intensity and workload), work risk (the pressure caused by the risk of work), and work development (the pressure caused by individuals’ feelings of job security and promotion). The higher the individual score, the greater the individual pressure. Validity analysis of work stress dimension was tested by exploratory factor analysis and confirmatory factor analysis. It showed that the model indicators: the KMO value is 0.954, greater than 0.8, which is suitable for factor analysis (21). The variance interpretation rate values of the three factors are 16.29% respectively, and the cumulative variance interpretation rate after rotation is 76.962% > 50%. It indicates that the information content of 76.962 of the 14 items in total could be extracted from the three factors, and generally more than 50% was acceptable. It can be considered that the measurement tool has good structural validity. In addition, the Cronbach’s α coefficient of this measurement was 0.958.

#### Risk perception

The COVID-19 risk perception scale developed by Cui Xiaoqian et al. (22) is a 9-item scale designed to measure one’s subjective feelings of risks. The scale contains three dimensions, namely, severity (severity of the epidemic), susceptibility (risk of infection), and controllability (the epidemic can be controlled). The participants responded on a five-point Likert scale ranging from 1 (strongly disagree) to 5 (strongly agree). The reliability analysis results showed that the Cronbach coefficient was 0.824. This indicates a good reliability coefficient of the scale. In addition, confirmatory factor analysis showed that the model fitting indices also met the standard range, GFI = 0.982, CFI = 0.972, RMSEA = 0.062, indicating good validity.

### Data analysis

Data were analyzed using SPSS 25.0 software and MS Excel. Descriptive statistics were used to analyze the sample characteristics. The psychological status of each dimension (continuous variable) for the police officers was expressed as the mean ± standard deviation (X ± S). One-way analysis of variance (ANOVA) was performed for data comparison among groups. Linear regression was used to analyze the factors affecting the psychological status of police officers. Statistical significance was determined at the 0.05 significance level.

## Results

Among the samples, there were 206 males, accounting for 68.21% and 31.79% were females. The majority of participants were 26–35 years old, accounting for 36.75%. Nearly 68.87% of civilian police are married. A total of 52.65% of police officers had been in the position for more than 4 years. The civilian police who gained a bachelor’s degree account for more than 50%. Approximately two-thirds of police officers have work experience in COVID-19 epidemic prevention. A total of 38.81% of them spent more than 8 h per week on epidemic prevention work (Table 1).

**Table 1:** Descriptions of the participants (*N* = 302).

Variables	Categories	Number	Percentage (%)
Gender	Male	206	68.21
	Female	96	31.79
Age (years)	18–25	37	12.25
	26–35	111	36.75
	36–45	69	22.85
	46–55	67	22.19
	>55	18	5.96
Marital status	Unmarried	79	26.16
	Married	208	68.87
	Divorce	15	4.97
Years of working	Below 1 year	58	19.21
	1–3 year	46	15.23
	4–5 year	39	12.91
	6-10 year	61	20.20
	>10 year	98	32.45
Education	High school and below	49	16.23
	Junior college	81	26.82
	Bachelor degree	163	53.97
	Master degree or above	9	2.98
Rank	Section member	71	23.51
	Deputy section chief	61	20.2
	Chief of section	26	8.61
	Deputy and above	15	4.97
	Others	129	42.72
Specific post	Security police	52	17.22
	Criminal police	46	15.23
	Constable police	59	19.54
	Community police	65	21.52
	Internal work police	80	26.49
Participate in COVID-19 epidemic prevention work	Yes	219	72.52
	No	83	27.48
Weekly working hours for epidemic prevention work	≤8	134	61.19
	9–16	48	21.92
	17–31	16	7.31
	≥32	21	9.59

According to the detection criteria of SCL-90 (23), the overall symptom severity distribution is shown in Table 2. The results showed that 37.75% of police officers scored 160 or above, 26.82% scored ≥ 2, and 38.74% of police officers scored ≥ 43 in positive items. In the 10 dimensions of the SCL-90 subdivision, the positive items ranked from high to low were obsessive–compulsive, depression, interpersonal sensitivity, hostility, somatization, paranoia, anxiety, psychosis, and phobia.

**Table 2:** Positive detection of the mental health status of police officers.

Dimension	Criteria	Number	Percentage (%)
Total score	≥160	114	37.75
Overall average score	≥2	81	26.82
Number of positive items	≥43	117	38.74
Obsessive–compulsive	≥2	128	42.38
Depression	≥2	97	32.12
Interpersonal sensitivity	≥2	92	30.46
Hostility	≥2	90	29.80
Somatization	≥2	84	27.81
Paranoia	≥2	77	25.50
Anxiety	≥2	75	24.83
Psychosis	≥2	62	20.53
Phobia Anxiety	≥2	50	16.56

Meanwhile, this study was compared with two norms’ results in Table 3. First, compared with the medical staff norm (16). The results showed that the SCL-90 dimension scores of police officers were significantly (*P* < 0.001) higher than those of the medical staff norm. This means that the psychological problems of the police are more serious than those of medical staff. Second, this study also compared with the military SCL-90 norm reported by Wang Huanlin et al. (24). Only one dimension (interpersonal sensitivity) of scoring among the police officers was lower than the military norm. On the other hand, the police offices had higher scores in the depression, somatization and psychosis dimensions than the military norm.

**Table 3:** SCL-90 scores of police officers compared with national and military norms (mean ± SD).

Dimension	SCL-90 (*n* = 302)	Medical staff norm (*n* = 395)	*t*	Military norm (*n* = 19,662)	*t*
Somatization	1.66 ± 0.76	1.39 ± 0.48	8.448^***^	1.55 ± 0.57	5.139^***^
Obsessive–compulsive	1.95 ± 0.83	1.58 ± 0.57	8.225^***^	1.77 ± 0.60	−1.687
Interpersonal sensitivity	1.72 ± 0.80	1.33 ± 0.50	1.925	1.78 ± 0.61	3.429^**^
Depression	1.76 ± 0.80	1.42 ± 0.54	6.473^***^	1.64 ± 0.60	3.616^***^
Anxiety	1.63 ± 0.77	1.31 + 0.49	7.446^***^	1.52 ± 0.52	1.385
Hostility	1.67 ± 0.78	1.36 ± 0.49	4.945^***^	1.62 ± 0.62	1.900
Phobia anxiety	1.40 ± 0.66	1.18 ± 0.38	5.763^***^	1.35 ± 0.45	−1.637
Paranoia	1.61 ± 0.75	1.24 ± 0.44	4.678^***^	1.67 ± 0.63	0.685
Psychosis	1.53 ± 0.71	1.22 ± 0.42	7.800^***^	1.51 ± 0.50	3.309^**^

^*^p < 0.05; ^**^p < 0.01; ^***^p < 0.001.

### Correlation analysis of mental health status, work stress, and risk perception of COVID-19 among police officers

To explore the relationship among mental health status, work stress and risk perception of COVID-19 among police officers, we conducted a correlation analysis in this study. Our results (Table 4) showed that the total SCL-90 score and its subdimensions were positively correlated with work stress and epidemic risk perception. The results indicated that work stress the police faced is likely to induce the worse the psychological condition. Accordingly, the high risk perception of COVID-19 epidemic could increase their chances of mental distress.

**Table 4:** Correlation matrix for mental health, work stress and risk perception.

	Total score	Somatization	Obsessive-compulsive	Interpersonal sensitivity	Depression	Anxiety	Hostility	Phobia	Paranoia	Psychosis
Work stress	0.569^***^	0.566^***^	0.577^***^	0.533^***^	0.569^***^	0.532^***^	0.528^***^	0.431^***^	0.549^***^	0.487^***^
Work load	0.533^***^	0.544^***^	0.520^***^	0.504^***^	0.525^***^	0.506^***^	0.499^***^	0.426^***^	0.496^***^	0.451^***^
Work risk	0.526^***^	0.538^***^	0.546^***^	0.485^***^	0.511^***^	0.490^***^	0.481^***^	0.399^***^	0.511^***^	0.448^***^
Career development	0.542^***^	0.503^***^	0.559^***^	0.507^***^	0.570^***^	0.497^***^	0.504^***^	0.381^***^	0.539^***^	0.470^***^
Risk Perception	0.358^***^	0.336^***^	0.384^***^	0.371^***^	0.357^***^	0.331^***^	0.326^***^	0.262^***^	0.335^***^	0.301^***^
Severity	0.255^***^	0.255^***^	0.284^***^	0.263^***^	0.268^***^	0.229^***^	0.217^***^	0.171^**^	0.208^***^	0.196^***^
Susceptibility	0.347^***^	0.336^***^	0.338^***^	0.337^***^	0.308^***^	0.341^***^	0.314^***^	0.306^***^	0.368^***^	0.307^***^
Controllability	0.285^***^	0.242^***^	0.325^***^	0.318^***^	0.302^***^	0.253^***^	0.279^***^	0.180^**^	0.265^***^	0.247^***^

^*^p < 0.05; ^**^p < 0.01; ^***^p < 0.001.

### Exploring the moderated effect model among police officers

Hierarchical regression analysis was conducted with work stress as the independent variable, gender, age, marriage, years of work, education, and epidemic prevention work as the control variables, and mental health (SCL-90) as the outcome variable, and we further tested the effect of risk perception. As shown in Table 5, the R^2^ of Model 3 was 0.39, which was greater than 0.19, indicating that the model has good explanatory power. Moreover, work stress was found to be positively associated with mental health (*p* < 0.001). Interaction item (work stress × risk perception) did not show significant (*t* = 1.32, *p* = 0.19 > 0.05).

**Table 5:** Work stress on mental health among the police: the moderating role of risk perception.

	Model 1				Model 2				Model 3			
	*β*	SE	*t*	*p*	*β*	SE	*t*	*p*	*β*	SE	*t*	*p*
Constant	-	28.56	−3.26	*p* < 0.001	-	28.39	−3.17	*p* < 0.001	-	28.94	−3.39	*p* < 0.001
Gender	−0.03	7.02	−0.68	0.50	−0.03	6.97	−0.70	0.49	−0.04	7.01	−0.85	0.40
Age	0.13	3.99	1.96	0.05	0.14	3.96	1.99	0.05	0.12	3.99	1.98	0.04
Marital status	0.22	7.84	3.51	*p* < 0.001	0.23	7.81	3.70	*p* < 0.001	0.24	7.82	3.81	*p* < 0.001
Years of working	0.11	2.78	1.65	0.10	0.10	2.76	1.62	0.11	0.10	2.76	1.50	0.13
Education	0.10	4.77	1.73	0.08	0.11	4.74	1.85	0.07	0.12	4.78	2.02	0.04
Rank	0.01	2.26	0.25	0.80	0.00	2.26	−0.02	0.98	0.00	2.26	−0.07	0.95
Post	0.05	1.93	0.98	0.33	0.05	1.92	1.02	0.31	0.06	1.91	1.04	0.30
Prevention work	0.01	7.68	0.22	0.83	−0.01	7.77	−0.21	0.83	−0.01	7.78	−0.10	0.92
Work stress	0.53	3.10	10.57	*p* < 0.001	0.46	3.69	7.63	*p* < 0.001	0.48	3.81	7.74	*p* < 0.001
Risk perception					0.13	4.18	2.25	0.03	0.13	4.17	2.27	0.02
Work stress*risk perception									0.06	2.86	1.32	0.19
*R^2^*	0.36				0.38				0.39			
Adjusted *R^2^*	0.35				0.36				0.37			
*F*	*F*(10,291) = 17.49 *p* < 0.001				*F*(11,290) = 16.58 *p* < 0.001				*F*(12,289) = 15.40 *p* < 0.001			

## Discussion

### Mental health, work stress, and epidemic risk cognition of Wuhan police

The results of this study showed that the positive detection rate of mental health was 37.75%, indicating that more than one-third of the police officers had poor mental health. The mental health level of Wuhan police is significantly higher than the normal level of medical staff, which may be associated with higher levels of work overload and occupational stress (25). In particular, they fought hard against the COVID-19 epidemic war at the front line. Exposure to risk of infection and unprecedented tasks increased their likelihood of impaired mental illness. This group may face higher job burnout and emotional exhaustion, which exacerbates negative mental health conditions.

In the present study, we also found that the mental health level of unmarried police officers was better than that of married police officers (β = 0.24, *p* < 0.001). Obviously, for married cops, police family cohesion becomes the key factor affecting mental health status (26). However, the special nature of police work means that they are often on duty, and their hours are not consistent with the lives of their families. Weak cohesion is concretely reflected in a lack of participation in family events. Without a doubt, it will inevitably lead to the chances of their physical and mental fatigue (27).

The results of this study also show that work stress has a significant positive impact on police officers’ mental health (β = 0.48, *p* < 0.001). The higher the work stress score was, the higher the total SCL-90 score. This result is in line with previous research (28). The specific reasons may be as follows: (1) During the epidemic, police officers were assigned more duties compared with usual times, such as patrolling residential areas, enforcing compliance on restriction of movements, and providing supplementary screening for confirmed COVID-19 cases. This has led to additional special work by police officers. Previous studies have shown that police officers will suffer from work stress due to long working hours and insufficient off-duty time; thus, these potential risk factors will make police officers more prone to depression, anxiety and other symptoms (29). (2) Police officers have poor stress self-management. Moreover, they are generally not provided with practical tools or strategies to help them regulate their negative thoughts and emotions. The inability to effectively alleviate psychological stress has its most threatened consequences in sustaining their resilience during the influenza pandemic (30). As a result, police were in a state of psychological stress for a long time and could not fully utilize their own coping resources for self-emotional regulation.

Another finding in this study was that mental health was positively correlated with epidemic risk perception, which was consistent with recent studies (31–33). Risk perception drives negative emotions and mental health problems (34). Teufel et al. observed a similar trend in their survey data (35); specifically, a higher perception of risk will increase the likelihood of higher levels of negative emotions. The police officers’ perception of risk uncertainty influenced their emotional response in specific hazard characteristics (13). Perceived hazards surrounding COVID-19 can again lead to psychological distress and stress symptoms (36). These findings suggest that perceptions of risk could amplify the social risk impact of the epidemic. The affect evoked by misinformation disclosure disrupts people’s cognition and judgment toward threats. Therefore, police administration should endorse problem-solving and emotion regulating strategies against COVID-19 in advance, improving the psychological resilience of police officers and reducing their risk perception to avoid more serious consequences.

## Limitation

This study has several limitations. First, we did not collect biological characteristics such as physical health and obesity data. These factors may affect the mental health of police officers and lead to potential residual confounding biases. Second, the participants were all from Wuhan, Hubei Province. Future studies should validate the findings using different samples of front-line workers from other provinces. Third, this cross-sectional study did not precisely reflect the dynamic mental health status of police officers during the COVID-19 epidemic. Thus, a longitudinal study design could be conducted for future research.

## Conclusion

In this study, the results showed that front-line police officers tended to show a higher prevalence of symptoms of mental disorders during the COVID-19 pandemic. The findings highlighted that age, marital status, and education level are important demographic factors affecting the mental health of the police. The greater the work stress and risk perception of police officers, the greater the negative impact on mental health. Furthermore, it is necessary to attach importance to the improvement and cultivation of consciousness in mental health screening among police officers. Policy-makers and social psychological health care organizations should establish an internal mental health problem handling team and inform innovative stress coping strategies to effectively promote police officers’ mental health.

## Data availability statement

The original contributions presented in the study are included in the article/supplementary material, further inquiries can be directed to the corresponding author.

## Ethics statement

The studies involving human participants were reviewed and approved by the Ethics Committee of Wuhan City College. The participants provided their written informed consent to participate in this study.

## Author contributions

JW contributed to conception and data curation and was responsible for writing original draft preparation and revising it critically. DL took responsibility for formal analysis and review and editing. QW contributed to project administration, interpretation of data, organization, and coordination. MX and JX were responsible for administrative, technical, or material support and supervision. XY supported participant recruitment and data analysis and interpretation. All authors contributed to the article and approved the submitted version.

## Funding

This research was supported by Natural Science Fund of Hubei Province (2022CFB365).

## Conflict of interest

The authors declare that the research was conducted in the absence of any commercial or financial relationships that could be construed as a potential conflict of interest.

## Publisher’s note

All claims expressed in this article are solely those of the authors and do not necessarily represent those of their affiliated organizations, or those of the publisher, the editors and the reviewers. Any product that may be evaluated in this article, or claim that may be made by its manufacturer, is not guaranteed or endorsed by the publisher.

## References

[1] Stogner J Miller BL McLean K. Police stress, mental health, and resiliency during the COVID-19 pandemic. Am J Crim Justice. (2020) 45:718–30. doi: 10.1007/s12103-020-09548-y, PMID: 32837167 PMC7319488

[2] Zhan W Jia Z. Research on prevention and control of occupational safety risks of police under the background of novel coronavirus epidemic. J Jiangsu Police Coll. (2021) 36:84–90. doi: 10.3969/j.issn.1672-1020.2020.05.012

[3] Jenkins SR. Coping and social support among emergency dispatchers: hurricane Andrew. J Soc Behav Pers. (1997) 12:201–16.

[4] Yuxuan L Jian Z. Research on coping strategies of psychological problems of community police under the background of prevention and control of novel coronavirus pneumonia. J Peoples Police Univ China. (2022) 38:85–90. doi: 10.3969/j.issn.1007-1784.2022.02.005

[5] Yong T Zhen W Yipeng Y Wen X Jingting H. Mental health status and influence of prison police under the prevention and control of novel coronavirus pneumonia epidemic. Henan J Prev Med. (2021) 22:32. doi: 10.13515/j.cnki.hnjpm.1006-8414.2021.10.006

[6] Zhu X Xia M Li S Hu Y Guo X. Mental status and psychological needs of Chinese police officers in a highly Impacted City during the COVID-19 pandemic. Int J Ment Health Promot. (2020) 22:e011097. doi: 10.32604/IJMHP.(2020) 011097

[7] Mengsheng C Qiang T. A study on the psychological cognition and law enforcement behavior of police under the COVID-19 epidemic−based on a statistical analysis of 1075 questionnaires nationwide. J Jiangsu Police Coll. (2020) 35:8. doi: 10.13515/j.cnki.hnjpm.1006-8414.2021.10.006

[8] Zhang YP Chu LF Zhuang KC Yang YW Zhang LP Xu ZH. Study on the stress, anxiety and depression of medical staff and its influencing factors under the COVID-19 epidemic. Modern Prev Med. (2021) 48:6.

[9] Lazarus RS Folkman S. Appraisal, coping, health status, and psychological symptoms. J Pers Soc Psychol. (1986) 50:571–9. doi: 10.1037/0022-3514.50.3.571 3701593

[10] Nielson KR Zhang Y Ingram JR. The impact of COVID-19 on police officer activities. J Crime Justice. (2022) 82:101943. doi: 10.1016/j.jcrimjus.2022.101943, PMID: 36247349 PMC9549130

[11] Violanti JM. Police organizational stress: the impact of negative discipline. Int J Emerg Ment Health. (2011) 13:31–6. PMID: 21957755

[12] Gierlach E Belsher BE Beutler LE. Cross-cultural differences in risk perceptions of disasters. Risk Anal. (2010) 30:1539–49. doi: 10.1111/j.1539-6924.2010.01451.x, PMID: 20626692

[13] Caserotti M Girardi P Rubaltelli E Tasso A Lotto L Gavaruzzi T. Associations of COVID-19 risk perception with vaccine hesitancy over time for Italian residents. Soc Sci Med. (2021) 272:113688. doi: 10.1016/j.socscimed.2021.113688, PMID: 33485215 PMC7788320

[14] Lindell MK Hwang SN. Households' perceived personal risk and responses in a multihazard environment. Risk Anal. (2010) 28:539–56. doi: 10.1111/j.1539-6924.2008.01032.x 18419668

[15] Chen Y Tan X Xing C Zheng J. How healthcare workers respond to COVID-19: the role of vulnerability and social support in a close relationships defense mechanism. Acta Psychol. (2021) 221:103442. doi: 10.1016/j.actpsy.2021.103442, PMID: 34717255 PMC8549441

[16] Wu Q Li D Yan M Li Y. Mental health status of medical staff in Xinjiang Province of China based on the normalisation of COVID-19 epidemic prevention and control. Int J Disaster Risk Reduct. (2022) 74:102928. doi: 10.1016/j.ijdrr.2022.102928, PMID: 35368428 PMC8958729

[17] Pappa S Ntella V Giannakas T Giannakoulis VG Papoutsi E Katsaounou P. Prevalence of depression, anxiety, and insomnia among healthcare workers during the COVID-19 pandemic: a systematic review and meta-analysis. Brain Behav Immun. (2020) 88:901–7. doi: 10.1016/j.bbi.2020.05.026, PMID: 32437915 PMC7206431

[18] Rosen CS Drescher KD Moos RH Finney JW Murphy RT Gusman F. Sixand tenitem indexes of psychological distress based on the Symptom Checist-90[J]. Assessment. (2000) 7:103–11. 10868247 10.1177/107319110000700201

[19] Hui X Wenli Q Feng W Lei F Lijuan Y. A survey on the psychological status of health care workers at county level in Hubei province under COVID-19. Psychol Monthly. (2020) 18:33–5. doi: 10.19738/j.cnki.psy.2020.18.011

[20] Wen L. Research on the relationship between job stress and job performance of grassroots police. Shanghai:East China University of Political Science and Law (2020).

[21] Li C Xin L. Research on evaluation method of reliability and validity of questionnaire. China Health Stat. (2008) 5:541–4.

[22] Xiaoqian C Yanhua H Siyu T Kaisheng F Yurong T Ning N. Reliability and validity test and application of the COVID-19 risk perception scale: an empirical study based on big data samples. J Public Health China. (2021) 37:1086–9.

[23] Xiangdong W Xilin W Hong M. Manual of the mental health rating scale. Chinese. J Ment Health. (1999) doi: 10.3760/j:issn:1006-7884.1999.01.012

[24] Wang Huanlin SJ Haiying Y Shi Jianan LN Chen Jijun MG Zou Huagen BIY Ming C. Establishment and result analysis of the norm of self-rating symptom scale for Chinese soldiers. Chin J Psychiatry. (1999) 1:37–9.

[25] Duxbury L Halinski M. It's not all about guns and gangs: role overload as a source ofstress for male and female police officers. Polic Soc. (2017) 28:930–46. doi: 10.1080/10439463.2017.1342644

[26] Agrawal M Mahajan R. The relationship between family cohesion, family-work conflict, enrichment and psychological health of Indian police. Policing. (2022) 45:794–811. doi: 10.1108/PIJPSM-02-2022-0028

[27] Masoudnia E. The relationship between deficiency of family cohesion and prevalence of physical and mental fatigue among Yazd province police families. J Milit Med. (2017) 19:306–14.

[28] Kelley DC Siegel E Wormwood JB. Understanding police performance under stress: insights from the biopsychosocial model of challenge and threat. Front Psychol. (2019) 10:1800. doi: 10.3389/fpsyg.2019.01800, PMID: 31447738 PMC6696903

[29] Queirós C Passos F Bártolo A Faria S Fonseca SM Marques AJ. Job stress, burnout and coping in police officers: relationships and psychometric properties of the organizational police stress questionnaire. Int J Environ Res Public Health. (2020) 17:17. doi: 10.3390/ijerph17186718, PMID: 32942672 PMC7557776

[30] McCraty R Atkinson M. Resilience training program reduces physiological and psychological stress in police officers. Glob Adv Health Med. (2012) 1:44–66. doi: 10.7453/gahmj.2012.1.5.013, PMID: 27257532 PMC4890098

[31] Yan J Kim S Zhang SX Foo MD Alvarez-Risco A Del-Aguila-Arcentales S. Hospitality workers' COVID-19 risk perception and depression: a contingent model based on transactional theory of stress model. Int J Hosp Manag. (2021) 95:102935. doi: 10.1016/j.ijhm.2021.102935, PMID: 36540684 PMC9756832

[32] Violanti JM Charles LE McCanlies E Hartley TA Baughman P Andrew ME. Police stressors and health: a state-of-the-art review. Policing. (2017) 40:642–56. doi: 10.1108/PIJPSM-06-2016-0097, PMID: 30846905 PMC6400077

[33] Garbarino S Cuomo G Chiorri C Magnavita N. Association of work-related stress with mental health problems in a special police force unit. BMJ Open. (2013) 3:e002791. doi: 10.1136/bmjopen-2013-002791 PMC3717472 23872288

[34] Leppin A Aro AR. Risk perceptions related to SARS and avian influenza: theoretical foundations of current empirical research. Int J Behav Med. (2009) 16:7–29. doi: 10.1007/s12529-008-9002-8, PMID: 19214752 PMC7090865

[35] Teufel M Schweda A Dörrie N Musche V Hetkamp M Weismüller B. Not all world leaders use twitter in response to the COVID-19 pandemic: impact of the way of Angela Merkel on psychological distress, behaviour and risk perception. J Public Health. (2020) 42:644–6. doi: 10.1093/pubmed/fdaa060, PMID: 32393966 PMC7239128

[36] Brooks SK Webster RK Smith LE Woodland L Wessely S Greenberg N. The psychological impact of quarantine and how to reduce it: rapid review of the evidence. Lancet. (2020) 395:912–20. doi: 10.1016/S0140-6736(20)30460-8, PMID: 32112714 PMC7158942

